# Supplementary material for: The genome sequence of the Clouded Buff, Diacrisia sannio (Linnaeus, 1758) (Lepidoptera: Erebidae)
Source: Wellcome Open Res. 2026 Jan 23;11:54. [Version 1] doi: 10.12688/wellcomeopenres.25823.1 (PMC13173286; doi:10.12688/wellcomeopenres.25823.1)
Supplement: Peer review history

# Reviewer report: Reviewer response for version 1

**DOI:** 10.21956/wellcomeopenres.28424.r146930

**Published:** 2026-05-13

**Authors:** Annabel Whibley

**Affiliations:** 1 Grapevine Improvement, Bragato Research Institute, Lincoln, New Zealand; 2 The University of Auckland, Auckland, New Zealand

Lucek and colleagues report a genome assembly of the Clouded Buff, *Diacrisia sannio,* with each haplotype reporting 31 pseudochromosomes (30 autosomes plus Z) and each containing >99.8 of the haplotype assembly lengths. The work was carried out as part of Project Psyche and the methods and reporting follow standard templating. I find the work to be systematically and comprehensively described, using contemporary and appropriate methods. The resulting assemblies are of excellent quality, supported by   strong evaluation statistics (each haplotype assembly reaches 6.C.66). No genome annotation or RNAseq data collection is reported at this time. Public data links and metadata are fully available. 

 Minor comment: the repetition of “mesophilic” in the opening paragraph feels unnecessary. Would “Diacrista sannio inhabits mesophilic ....” do the same work or is there extra information conveyed by underscoring the ecological niche of the species is also mesophilic?

Are sufficient details of methods and materials provided to allow replication by others?

Yes

Is the rationale for creating the dataset(s) clearly described?

Yes

Are the datasets clearly presented in a useable and accessible format?

Yes

Are the protocols appropriate and is the work technically sound?

Yes

Reviewer Expertise:

Genomics, Bioinformatics

I confirm that I have read this submission and believe that I have an appropriate level of expertise to confirm that it is of an acceptable scientific standard.

# Reviewer report: Reviewer response for version 1

**DOI:** 10.21956/wellcomeopenres.28424.r146924

**Published:** 2026-02-28

**Authors:** Nhat Tan Pham

**Affiliations:** 1 Adam Mickiewicz University in Poznań, Poznań, Poland

Authors describe the methods and results of genome assembly for the Clouded Buff, *Diacrisia sannio* (Linnaeus, 1758) (Lepidoptera: Erebidae). Two haplotypes were assembled using 56X PacBio data and Arima2 Hi-C data. The methods are logical and well-written. However, the method for identifying the Z chromosome has not yet been fully deciphered.

 “The Z chromosome was identified based on read coverage and BUSCO gene painting with Merian element”. No data is available to verify the coverage. The authors can also check homology with the Erebidae genome (e.g., *Hyphantria cunea*).

 The authors report two haplotypes from a genome assembly of *Diacrisia sannio*, using high-coverage (56X) PacBio HiFi and Hi-C sequences. Most of the assembly sequence was assigned to 30 autosomes and the Z chromosome *. *The authors also assembled the mitochondria and painted Merian elements in each chromosome. The method and results were well written, but a minor revision to the results is recommended to clarify how the authors identify the Z chromosome.

 In detail, the authors stated, “The Z chromosome was identified based on read coverage and BUSCO gene painting with Merian elements.” However, I could not find information on coverage. It can also be based on homology with a published Erebidae genome (eg. *Anticarsia gemmatalis*). Addressing this minor issue will strengthen the paper.

Are sufficient details of methods and materials provided to allow replication by others?

Yes

Is the rationale for creating the dataset(s) clearly described?

Yes

Are the datasets clearly presented in a useable and accessible format?

Yes

Are the protocols appropriate and is the work technically sound?

Yes

Reviewer Expertise:

genome assembly, Lepidoptera

I confirm that I have read this submission and believe that I have an appropriate level of expertise to confirm that it is of an acceptable scientific standard, however I have significant reservations, as outlined above.

# Response

**Published:** 2026-02-28

**Authors:** Tree of Life Team Sanger

**Affiliations:** Wellcome Trust Sanger Institute, UK

We thank the peer reviewer for reviewing this data note and for the suggestion to clarify how the Z chromosome was identified. Read coverage is shown in the BlobToolKit GC–coverage plot (Figure 7) and can be explored further in the linked interactive BlobToolKit viewer. In this assembly, coverage is broadly similar across chromosome-scale scaffolds, as expected for a homogametic (male, ZZ) individual. The Z chromosome was assigned using BUSCO Merian-element painting (Figure 4), where one chromosome is painted predominantly as the Merian Z element (MZ). Given this unambiguous MZ assignment, we did not perform an additional homology-based check against other erebid assemblies.

# Reviewer report: Reviewer response for version 1

**DOI:** 10.21956/wellcomeopenres.28424.r146220

**Published:** 2026-02-14

**Authors:** Jayan Duminda M Senevirathna

**Affiliations:** 1 Uva Wellassa University, Badulla, Uva Province, Sri Lanka

The manuscript is well written and includes the required data and methodological details. However, several minor revisions are recommended to improve clarity and presentation.

 The abstract should be revised for conciseness and clarity. When describing the two haplotypes, the sentences are currently very similar and somewhat repetitive; these can be combined to improve readability and flow. In addition, the broader importance and implications of the genome analysis should be more clearly highlighted in the abstract to emphasize the scientific contribution of the study.

 Furthermore, in Table 2, consistency should be maintained when presenting fragment lengths (e.g., units, formatting, decimal places), as inconsistencies may affect clarity and professionalism.

 Addressing these minor issues will further strengthen the manuscript.

Are sufficient details of methods and materials provided to allow replication by others?

Yes

Is the rationale for creating the dataset(s) clearly described?

Yes

Are the datasets clearly presented in a useable and accessible format?

Yes

Are the protocols appropriate and is the work technically sound?

Yes

Reviewer Expertise:

Aquatic Biotechnology

I confirm that I have read this submission and believe that I have an appropriate level of expertise to confirm that it is of an acceptable scientific standard.
